# Cerebrospinal fluid microbiome revisited: no evidence of resident bacteria in archived samples

**DOI:** 10.1128/spectrum.01308-25

**Published:** 2025-08-26

**Authors:** Angela Ward, Heekuk Park, Bin Cheng, Dwayne Seeram, Kiran T. Thakur, Anne-Catrin Uhlemann, James M. Noble, Panos N. Papapanou

**Affiliations:** 1 Division of Periodontics, Section of Oral, Diagnostic and Rehabilitation Sciences, College of Dental Medicine, Irving Medical Center, Columbia University 21611, New York, New York, USA; 2 Division of Infectious Diseases, Department of Medicine, Irving Medical Center, Columbia University 171475, New York, New York, USA; 3 Microbiome & Pathogen Genomics Core, Vagelos College of Physicians and Surgeons, Irving Medical Center, Columbia University 21611, New York, New York, USA; 4 Department of Biostatistics, Mailman School of Public Health, Irving Medical Center, Columbia University 189434, New York, New York, USA; 5 Department of Neurology, Vagelos College of Physicians and Surgeons, Irving Medical Center, Columbia University 171461, New York, New York, USA; 6 Taub Institute for Research on Alzheimer’s Disease and the Aging Brain and Gertrude H. Sergievsky Center, Irving Medical Center, Columbia University 171553 , New York, New York, USA; Nanchang University, Nanchang, Jiangxi, China

**Keywords:** cerebrospinal fluid, dementia, infection, periodontitis, 16s rRNA sequencing

## Abstract

DNA from oral bacteria has been detected in the cerebrospinal fluid (CSF) of patients with Alzheimer’s disease and related dementias (AD/ADRD). We hypothesized that examination of archived CSF samples from donors with variable cognitive status would reveal evidence of a resident microbiome. 176 CSF samples harvested from community-dwelling individuals (77% between 61 and 80 years old) were analyzed; 57% originated from donors with impaired cognitive status. DNA was extracted after adding microbial spike-in controls, and libraries were prepared and sequenced on an Illumina-MiSeq platform. 16S rRNA sequences were processed, and a taxonomic classification was performed. Spike-in bacteria were consistently detected, and *Streptococcus pneumoniae* was found in a positive control sample from a patient with bacterial meningitis. However, very few reads mapping to other bacterial taxa were detected across samples, suggesting a negligible bacterial content consistent with occasional contamination or sequencing errors. CSF is a privileged, sterile environment that does not harbor a resident microbiome in elderly people with various morbidities, including AD/ADRD.

**IMPORTANCE:**

Recent reports have suggested that DNA from oral bacteria has been found in the cerebrospinal fluid (CSF) of patients with Alzheimer's disease and related dementias (AD/ADRD). We hypothesized that examination of archived CSF samples from donors with variable cognitive status would reveal evidence of a resident microbiome. We thus analyzed 176 CSF samples harvested from community-dwelling individuals including donors with impaired cognitive status. While our findings suggested the presence of occasional bacterial contamination, they provided no evidence of a resident microbiome. We thus conclude that the CSF is indeed a privileged, sterile environment that does not harbor a resident microbiome in elderly people with various morbidities including AD/ADRD.

## INTRODUCTION

The role of the human microbiome in homeostasis and preservation of health (1), but also its involvement in various pathological conditions (2, 3), is increasingly recognized. Lately, the potential contribution of microbial exposures to Alzheimer’s disease and related dementias (AD/ADRD) has gained increasing attention (4, 5). Recent research studies have focused on the putative role of the oral microbiome, along with the systemic inflammatory burden conferred by periodontitis, on the development of AD/ADRD (6, 7).

Periodontitis is a chronic inflammatory disease that results in progressive destruction of the tooth-supporting tissues, ultimately leading to tooth loss, impaired masticatory function, poor nutrition, and lower quality of life (8). Dysbiotic microbial plaques that accumulate on the tooth surfaces below the gingival margin provide the impetus for the sustained local inflammatory response encountered in periodontitis that results in tissue destruction (9). Epidemiologic studies have shown that periodontitis is associated with multiple comorbidities, but a role of the disease as an exposure that independently confers risk for extraoral pathology is biologically plausible (10, 11). The 2024 report of the Lancet Standing Commission on dementia prevention, intervention, and care (12) includes periodontitis as well as inflammation related to dental disease to the list of potential risk factors for dementia which, however, have not been unequivocally established with sufficient, high-level evidence.

Recent data suggest a substantial increase in the prevalence of severe periodontitis over the past 30 years, mainly due to population growth (13). The disease is more common in current smokers, and in people with diabetes, lower socioeconomic status, older age, and male sex (14).

Aside from case studies linking periodontitis with overt intracerebral infections including abscesses and ventriculitis (15, 16), plausible scientific questions have been raised about potential mechanisms linking periodontitis and cognitive impairment. Interestingly, associations have been reported between distinct oral microbiome signatures and cognitive function (17), as well as between periodontal bacterial dysbiosis and reduced levels of amyloid Aβ42 in the cerebrospinal fluid (CSF) of cognitively normal elderly (18). Furthermore, serum antibodies to periodontal bacteria were found to discriminate between individuals with and without AD (19). In a case-cohort study, our group reported an association of periodontitis with incident AD (20).

Experimental animal studies have shown that infection by *Porphyromonas gingivalis,* an established periodontal pathogen, results in the formation of amyloid plaques (21), promotes neurodegeneration and the formation of neurofibrillary tangles (22, 23), and activates microglia toward a proinflammatory and phagocytic phenotype (24, 25).

Furthermore, DNA from *P. gingivalis* has been detected in the CSF of people with AD (26, 27). Another research group examined multiple brain samples from AD patients and control individuals with normal brain histology and identified the presence of *P. gingivalis* DNA in both groups and DNA for the non-oral bacterium *Cloacibacterium normanense* exclusively in AD-affected regions (28).

These observations raise multiple questions related to a potentially unrecognized resident microbiome of the CSF, including whether (i) additional oral bacteria beyond *P. gingivalis*, or other bacteria of non-oral origin, may also be commonly present in the CSF; and (ii) entry of bacteria in the CSF occurs exclusively in people with AD/ADRD, or in the presence of other comorbidities, or even in people devoid of AD/ADRD pathologies or clinically manifest syndromes.

While the CSF has traditionally been regarded as a sterile fluid, where bacterial presence signifies a central nervous system infection such as meningitis, recent studies have reported a sparse presence of bacteria in the CSF, but have cautioned that the findings do not necessarily indicate the presence of a resident microbiome but rather sample contamination (29, 30). Nevertheless, other authors have argued that the olfactory and trigeminal nerves can serve as pathways that may allow bacteria and viruses to enter the brain, bypassing the blood-brain barrier or the blood-CSF barrier, and to contribute to pathological changes (31). A potential role for the gut microbiota in the disruption of the gut epithelial barrier, the blood-brain barrier, and the blood-CSF barrier has been suggested as well (32). Importantly, a distinction must be drawn between transient or occasional bacterial presence in the CSF—that most studies reporting positive findings seem to indicate—and a true microbiome that would suggest the presence of stable and functional bacterial communities.

To further elucidate the issue of whether the CSF is indeed a privileged, sterile environment, or a fluid that indeed harbors a resident microbiome, we examined the microbial content of archived CSF samples available through a CSF biobank at the Columbia University Medical Center. We hypothesized that examination of CSF samples from donors with different morbidities, including individuals with different levels of cognitive status, would reveal the presence of a resident CSF microbiome.

## MATERIALS AND METHODS

The study was conducted at the Columbia University Medical Center after approval by the Institutional Review Board (protocol # AAT1249).

### CSF sample provenance and storage

A total of 176 consecutively archived CSF samples available through the biobank of the Neurological Institute of New York were used. These samples had been obtained from community-dwelling individuals who had presented to the clinical practice or to cognitive aging research programs of the Institute for a variety of reasons, including neurological examination for the diagnosis of cognitive status. CSF sample donors had consented to have a lumbar puncture and had also agreed to allow further use of their CSF samples for research purposes. An additional sample originating from a patient diagnosed with bacterial meningitis was included as a positive control.

After collection, the samples were transported on ice to a common laboratory for end-stage processing. Depending on the primary reason for sample collection, samples were further treated according to one of two protocols. According to protocol A (*N* = 125), samples underwent a 2,000 × *g* centrifugation for 10 min at 4°C after collection. Samples processed according to protocol B (*N* = 51) were first centrifuged at 300 × *g* for 10 min, at 4°C, to first collect a pellet of mammalian cells, before being centrifuged a second time as per protocol A. In both groups of samples, the CSF supernatants were removed using a common sterile technique, and the remnant pellets were stored at −80°C until further processed for microbiome analysis in this study.

### DNA extraction and 16S rRNA gene sequencing

To maximize bacterial DNA extraction efficiency from the archived CSF samples, we used the QIAamp 96 Virus QIAcube HT kit (Qiagen, Germany). To further verify extraction efficiency, we added 1 µL of ZymoBIOMICS Spike-in Control I (High Microbial Load) to each sample, corresponding to approximately 1 × 10^6^ cells of *Imtechella halotolerans* and *Allobacillus halotolerans* per sample. This low concentration of spike-in bacteria was purposefully selected to preclude occluding any low-biomass signal in the sample. All extractions included both negative (water only) and positive controls (ZymoBIOMICS Microbial Community Standard; Zymo Research, CA, USA), which consists of defined bacterial communities.

Library preparation was carried out using the Zymo Quick-16S/V3V4 Plus NGS Library Prep Kit (Zymo Research) that employs the Equalase technique and allows precise amplification of the 16S rRNA gene using qPCR and facilitates its subsequent indexing. The libraries were then sequenced on an Illumina MiSeq platform using the v3 reagent kit (600 cycles) at a loading concentration of 8 pM, including a 20% phiX spike-in.

### Data processing

The 16S rRNA sequences were processed using the DADA2 pipeline (DADA2 1.12.1, R v4.1.0) (33). Demultiplexed FASTQ sequences were quality-filtered, trimmed, de-replicated, filtered for chimeric sequences, and amplicon sequence variant tables were generated. Taxonomic classification was undertaken using a native naïve RDP Bayesian classifier aligned against the Silva version 138 database (34). Subsequent to DADA2 pipeline processing, any mitochondrial reads were identified and removed, to ensure that only microbial genomic data were assessed.

Descriptive statistics were used to summarize the demographic information of the individual CSF sample donors. The data were integrated into R (version 4.1.0) using the phyloseq package (v1.36.0) (35) for analyzes. Based on α-diversity rarefaction, we applied a minimum cutoff of 7,500 counts for sample inclusion. To ensure data quality, sequences were filtered to retain only those with lengths between 400 and 450 base pairs. Additionally, a stringent abundance cutoff of 0.0001% was applied to remove extremely low-abundance taxa that could introduce noise into the analysis. Less abundant bacteria were grouped under the category “Other.”

Sequencing data for the study are publicly available through the NCBI Sequencing Read Archive (PRJNA1191488) after filtering off any human-derived sequences.

## RESULTS

Table 1 presents the basic demographic information of the donors of the 176 CSF samples, 55.1% of whom were male, and 76.7% were between 61 and 80 years old at the time of sample collection. The majority of the donors were White (55.7%), 11.9% were Hispanic, and 45% had at least a college-level education.

**TABLE 1:** Demographic information of the donors of the 176 CSF samples

		*N*	%
Gender	Male	97	55.1
	Female	79	44.9
Age (years)	42–60	27	15.3
	61–80	135	76.7
	81–95	14	8.0
Race	White	98	55.7
	Black	14	8.0
	Asian	2	1.1
	Other	19	10.8
	Not reported	43	24.4
Ethnicity	Not Hispanic	101	57.4
	Hispanic	21	11.9
	Not Reported	54	30.7
Education	Lower than High School	7	4.0
	High School	30	17.0
	College	33	18.8
	Higher than College	46	26.1
	Not Reported	60	34.1

Table 2 describes the occurrence of selected comorbidities and smoking history among the sample donors; 50% of the donors had hyperlipidemia, 45% had hypertension, and 12% were diagnosed with diabetes, while 5% and 32% of them, respectively, were current or former smokers.

**TABLE 2:** Selected comorbidities and smoking history among the sample donors

		*N*	%
Comorbidities	Hyperlipidemia	70	39.8
	Hypertension	79	44.9
	Diabetes	22	12.5
	Unknown	5	2.8
Smoking history	Current	9	5.1
	Former	56	31.8
	Never	110	62.5
	Unknown	1	0.6

Tables 3 to 5 describe the cognitive status of the donors based on clinical assessments (Table 3), on Mini-Mental State Exam (MMSE) scores (Table 4), and on CSF biomarker profiles generated using the Admark panel (Athena Diagnostics, Marlborough, MA, USA; Table 5). Based on clinical assessments, approximately 57% of the donors were diagnosed with either AD-related or non-AD-related dementia, 20% were diagnosed with mild cognitive impairment (MCI), and 23% were deemed to be cognitive normal (Table 3). MMSE scores were available from 115 donors and suggested that 52% of those with available data had some form of dementia (Table 4). One-third of the donors had a CSF biomarker profile consistent with a diagnosis of AD (Table 5).

**TABLE 3:** Cognitive diagnosis of the donors, based on clinical assessments

	*N*	%
Cognitively normal after clinical examination	10	5.7
Research participants; presumed cognitively normal	30	17.0
AD-related MCI	12	6.8
Non-AD-related MCI	24	13.6
AD-related dementia	77	43.8
Non-AD-related dementia	23	13.1

**TABLE 4:** Cognitive status of the donors based on Mini-Mental State Exam (MMSE) scores

	*n*	%
≥24 (normal cognition)	55	31.3
19–23 (mild dementia)	33	18.8
10–18 (moderate dementia)	25	14.2
≤9 (severe dementia)	2	1.1
No MMSE scores available	61	34.6

**TABLE 5:** Cognitive diagnosis of the donors based on CSF biomarker profiles^*d*^

Total *n* = 176	*n*	%
Consistent with AD^*a*^	58	33.0
Borderline^*b*^	40	22.7
Not consistent with AD^*c*^	26	14.8
Indeterminate	14	7.9
No biomarker profile available	38	21.6

^*a*^ Consistent with AD: p-Tau > 68 pg/mL and ATI < 0.8.

^*b*^ Borderline: p-Tau 54–68 pg/mL and/or ATI 0.8–1.2.

^*c*^ Not consistent with AD: p-Tau < 54 pg/mL and ATI > 1.2.

^*d*^ Produced using Admark panel (Athena Diagnostics) that assesses CSF Aβ42, tTau, pTau, and the Aβ42/total tau index (ATI).

**Fig 1:**
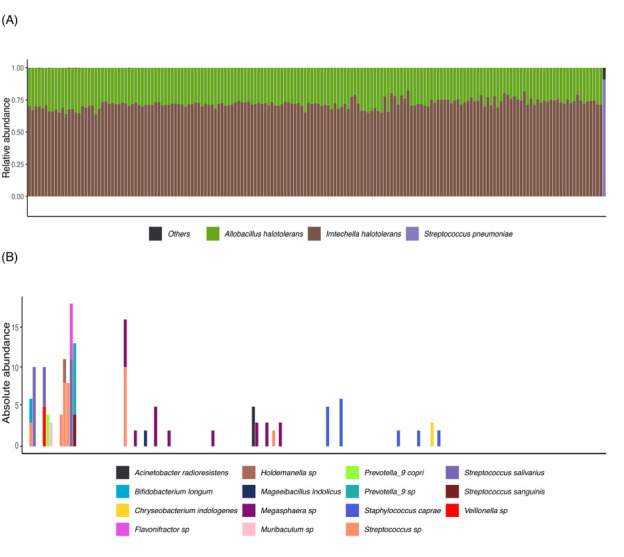
Relative and absolute abundance of bacterial taxa detected in CSF samples. Samples are arranged using barycentric distance-based clustering. (**A**) Relative abundance of bacterial taxa detected in the 174 successfully processed cerebrospinal fluid (CSF) samples, as well as in a positive control from a patient with bacterial meningitis (last sample at the far right), based on a stringent abundance cutoff of 0.0001%. The two spike-in bacteria, *A. halotolerans* and *I. halotolerans*, were the only taxa consistently detected across all samples, while *Streptococcus pneumoniae* was identified in the positive control sample. (**B**) Absolute abundance of bacterial taxa classified as “Other” based on an abundance cutoff of 0.0001%. A number of bacterial taxa were detected with fewer than 20 reads each, suggesting that they were occasional contaminants or sequencing errors rather than resident bacteria.

Of the 176 CSF samples sequenced, 174 yielded over 7,500 reads and were included in all downstream analyses. Both spike-in bacteria, *A. halotolerans* and *I. halotolerans*, were consistently detected across all samples (Fig. 1A). However, few or no reads mapping to other bacterial taxa were detected across all samples, suggesting a negligible burden of bacteria in the CSF samples analyzed. In contrast, in the positive control sample from the patient with clinically diagnosed bacterial meningitis, only *Streptococcus pneumoniae* was detected, as expected.

While bacterial read counts were very low after removal of the spike in controls, we further investigated the taxa that were filtered out due to low abundance in the samples (Fig. 1B). Using absolute abundance as a measure, we observed the presence of several bacterial taxa, but with bacterial counts consistently fewer than 20 reads. This extremely low count suggests that these taxa were not genuinely present in the CSF samples, but likely represent contaminants introduced during sample processing or sequencing errors.

## DISCUSSION

In this study, we used 16S rRNA gene sequencing to analyze the bacterial content of a substantial number of archived CSF samples obtained from donors with a variety of conditions to investigate whether presence of a resident microbiome is a routine finding. Of note, our work is responsive to the recently established consensus protocol and call for collaboration to explore the brain pathobiome in patients with mild cognitive impairment and Alzheimer’s disease (36), which explicitly encourages the study of CSF for the investigation of the brain microbiome. However, our findings revealed very low relative abundance by bacteria other than the imputed positive controls, suggesting minor sample contamination. Thus, we conclude that, barring conditions such as central nervous system infections, the CSF is a microbiologically privileged, sterile fluid.

As stated in the introduction, the impetus of the current work was provided partly by literature suggesting a possible contribution of infections to the etiopathogenesis of AD/ADRD, and more specifically by publications that reported frequent presence of both DNA and proteolytic enzymes originating from the periodontal pathogen *P. gingivalis*, in the CSF as well as in brain tissues of patients with Alzheimer’s disease (26, 27). This observation raised important questions, including whether DNA from additional oral or non-oral species can also be routinely recovered from CSF samples from donors with impaired cognitive ability, or from other individuals with comorbidities that may also negatively affect the blood-brain barrier and may facilitate transient entry of bacteria from multiple sources into the central nervous system. We thus embarked on an investigation of a collection of CSF samples of varying provenance for possible bacterial content using 16S rRNA gene sequencing. Should our findings indicate that the presence of bacterial DNA in the CSF is a common finding, we also aimed to trace the potential origin of the most abundant species by examining the donor’s oral and/or skin microbiota. Obviously, the planned subsequent steps of the project became unwarranted, given our finding of negligible bacterial yields that were suggestive of the presence of occasional contaminants. Notably, the analyzed CSF samples originated from a donor pool that encompassed a wide range of conditions, including health, current smoking, diabetes, cardiovascular disease, cognitive impairment, and other neurological conditions. Nevertheless, no evidence suggesting a resident CSF microbiome was observed. Importantly, the sample derived from a patient with bacterial meningitis harbored an abundance of *S. pneumoniae*, demonstrating the ability of our assays to detect the presence of bacterial DNA in the case of a true CNS infection.

Our work does not corroborate the findings by Dominy et al. (26, 27) of frequent presence of *P. gingivalis* DNA in the CSF of people with AD. Although approximately half of our samples originated from donors with AD-related MCI/dementia (Table 3), *P. gingivalis* DNA was not encountered in any of the samples, even when extremely low abundance signals were analyzed (Fig. 1B). This discrepancy could partly reflect differing assays for prokaryotic DNA detection, but possibly also differences in the timing of CSF collection in relation to disease state between the studies. However, given the fairly large number of CSF samples analyzed, temporal variation in the microbial content due to transient entry of bacteria in the CSF related to disease status should reasonably have been reflected in a subset of the samples, if present. Our findings are largely in agreement with recent publications that have studied potential bacterial presence in CSF samples originating from specific donor cohorts. Using metagenomic and meta-transcriptomic sequencing, a study (29) reported that CSF samples from healthy pregnant women do not harbor a detectable bacterial community. Likewise, two studies (37, 38) that analyzed CSF samples from patients with multiple sclerosis reported that they were generally free from bacterial DNA or suggested the presence of contaminants. In contrast, distinct CSF microbial signatures were detected in children with bacterial meningitis (39, 40), in patients with and without HIV with suspected CNS infection (41), as well as in children with hydrocephalus (30) or CSF shunt infections (42–44).

Our study is not without limitations. First, a subset of the CSF samples analyzed in this study (51 out of 176) had undergone a first centrifugation step to collect mammalian cells present in the CSF. Conceivably, the pellets collected during this first centrifugation that were not available for microbiome analysis could also have harbored bacterial cells. However, no systematic difference in bacterial signal, including that of low-abundance species, was observed between CSF samples subjected to the two centrifugation protocols. To further rule out this possibility, we analyzed available sequencing data from an additional 50 CSF samples not included in this study, originating from centrifugation pellets harvested according to the second protocol employed in our study, and again were unable to detect the presence of bacterial reads beyond the stipulated abundance threshold (data not shown). Second, our study was unable to explore the presence of bacterial outer membrane vesicles (BMVs) in the samples, which may act as infectious exposures but may be undetectable by approaches relying on prokaryotic DNA detection. Current technologies for BMV detection have been applied on blood/plasma samples but not on CSF (45). Third, our analyses are based on 16S rRNA sequencing, and more robust metagenomic sequencing methodologies might have yielded different findings. Fourth, the periodontal status of the sample donors was unknown. Notwithstanding the above limitations, our findings provide no evidence of a substantial or persisting translocation of oral or non-oral bacteria to the CSF, resulting in the presence of a resident microbiome.
